# Advances in the relationship between temporal muscle thickness and prognosis of patients with glioblastoma: a narrative review

**DOI:** 10.3389/fonc.2023.1251662

**Published:** 2023-09-13

**Authors:** Jinhai Tang, Zhenghao Dong, Junxiu Sheng, Ping Yang, Wanying Zhao, Juan Xue, Qizheng Li, Li Lv, Xiupeng Lv

**Affiliations:** ^1^ Department of Radiation Oncology, the First Affiliated Hospital of Dalian Medical University, Dalian, Liaoning, China; ^2^ Department of Thoracic Surgery, West China Hospital of Sichuan University, Chengdu, Sichuan, China; ^3^ Department of Pathology, the Second Affiliated Hospital of Dalian Medical University, Dalian, Liaoning, China

**Keywords:** glioblastoma, temporal muscle thickness, sarcopenia, frailty, prognosis, overall survival, progression-free survival

## Abstract

The most dangerous variety of glioma, glioblastoma, has a high incidence and fatality rate. The prognosis for patients is still bleak despite numerous improvements in treatment approaches. We urgently need to develop clinical parameters that can evaluate patients' conditions and predict their prognosis. Various parameters are available to assess the patient's preoperative performance status and degree of frailty, but most of these parameters are subjective and therefore subject to interobserver variability. Sarcopenia can be used as an objective metric to measure a patient's physical status because studies have shown that it is linked to a bad prognosis in those with cancers. For the purpose of identifying sarcopenia, temporal muscle thickness has demonstrated to be a reliable alternative for a marker of skeletal muscle content. As a result, patients with glioblastoma may use temporal muscle thickness as a potential marker to correlate with the course and fate of their disease. This narrative review highlights and defines the viability of using temporal muscle thickness as an independent predictor of survival in glioblastoma patients, and it evaluates recent research findings on the association between temporal muscle thickness and prognosis of glioblastoma patients.

## 1 Introduction

About 50% of all gliomas are glioblastomas (GBM), which are invasive original intracranial malignant tumors with a high rate of morbidity and mortality (1). The Stupp regimen is currently considered the preferred treatment for GBM and includes maximum surgical resection followed by radiation therapy combined with temozolomide (TMZ) chemotherapy (2). Despite effective intervention and treatment, the prognosis for GBM remains poor, with almost all patients progressing within one year (2).

Age, preoperative performance status, the location of the tumor, the degree of resection, adjuvant therapy, and genomic features are some of the factors that have been proven to affect the prognosis of GBM patients (3, 4). The patient's preoperative functional status is an important reference that affects prognosis and can be assessed clinically prior to surgery. However, the subjective assessment of the doctor affects the appraisal of the client's clinical status, leading to significant interobserver variability and a lack of objectivity in the clinical assessment (5). Therefore, there is an urgent need for objectively measurable parameters to estimate the degree of frailty of patients to improve the assessment of their prognosis. The assessment of skeletal muscle mass can be used as an objective parameter to determine the physical condition of the patient.

Sarcopenia is the term for the loss of skeletal muscle mass, and it is a key component of cancer-related cachexia as well as a crucial prognostic factor in surgical oncology (6, 7). The ability to determine sarcopenia using temporal muscle thickness (TMT) has been demonstrated to be a viable alternative to measuring skeletal muscle mass (8, 9). TMT may be utilized as an independent criterion for predicting frailty and survival in patients with brain tumors, according to various studies that have looked at the association between survival time and TMT in patients with brain metastases in recent years (10, 11).

Recently, the relationship between TMT and prognosis of GBM patients has become a hot topic of research, and it has been pointed out that TMT has the potential to be a standalone risk factor for a patient's prognosis for GBM, which can help to assess the survival of patients (12, 13), However, its conclusions are still controversial. Therefore, this paper reviews the latest research progress on the relationship between TMT and GBM prognosis, and discusses the impact of TMT on patients with GBM patients' prognoses and the prospect of clinical application.

## 2 Methods

### 2.1 Search strategy

The studies included for narration in this article were systematically searched in PubMed, Embase, and Medline by two authors (Jinhai Tang and Zhenghao Dong), with the last search updated on May 30, 2023. The keywords used in the search mainly included "Glioblastoma", "astrocytoma", "sarcopenia" and "temporal muscle". The search process also strictly followed the Preferred Reporting Items for Systematic Evaluation and Meta-Analysis(PRISMA) list (14). Non-original articles (i.e., review articles and meta-analyses), case reports and letters were excluded. In addition, additional studies that met our criteria were identified by manually searching for reviews on the topic or references to the original article. Details of the search strategy are listed in the supplementary material.

The studies were screened by title and abstract. Full texts were downloaded for review, if needed. Reference lists of extracted studies were also reviewed. The final selected studies were independently screened by three Authors(Jinhai Tang, Zhenghao Dong and Junxiu Sheng).

### 2.2 Inclusion and exclusion criteria

The following criteria had to be met for inclusion: (1) all subjects were glioblastomas; (2) the study design was a case-control or cohort study; (3) the study was related to temporal muscle thickness and prognosis of glioblastoma.

Studies that met one of the following criteria were excluded: (1) the subject of the study was not glioblastoma or temporal muscle thickness; (2) non-original papers such as conference papers, meta-analyses, or letters; (3) valid data such as OS, median temporal muscle thickness, etc., were unavailable; (4) the study was not written in English.

### 2.3 Data extraction

Data were extracted from two independent authors (Jinhai Tang and Zhenghao Dong). Details extracted from the included studies were as follows: first author's name, tumor type (primary GBM or progressive GBM), mean temporal muscle thickness, OS, PFS, HR (OS), 95% CI (OS), P value (OS). Disagreements between researchers were resolved by discussion and consensus. In case of disputes, an independent third-party investigator was responsible for resolving disagreements.

### 2.4 Search results

**Figure 1:**
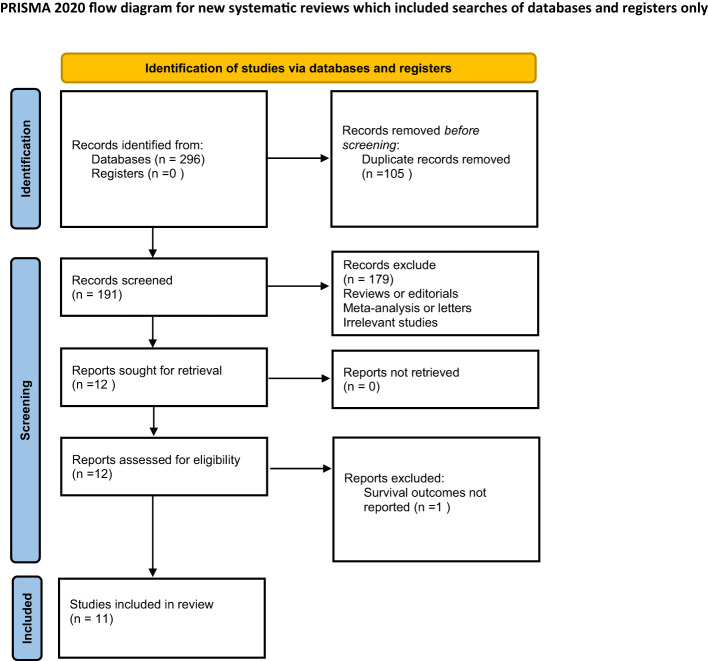
PRISMA flowchart of the selection of articles.

The systematic search retrieved a total of 296 records, including 105 duplicates and 191 original publications (**Figure 1**). All 191 unique studies and trials were screened for eligibility, after which 179 publications were excluded by reading titles and abstracts. Reasons for exclusion included conference abstract reports, case reports, literature reviews, and study topic discrepancies. The full text of 12 studies was evaluated, including 1 that did not explore in detail the relationship between temporal muscle thickness and glioblastoma prognosis. 11 studies that met the criteria were ultimately included for narration.

## 3 Sarcopenia and TMT

### 3.1 Definition and diagnosis of sarcopenia

Sarcopenia was first used to characterize the loss of muscle mass brought on by physiological aging in the old population. Sarcopenia is now understood to be a progressive systemic skeletal muscle condition with diminished skeletal muscle mass and hypofunction (15), Sarcopenia is now well understood as a result of ongoing study. The definition of sarcopenia as a syndrome characterized by age-related loss of skeletal muscle mass and loss of function, including loss of muscle strength and/or physical performance, was agreed upon by the European Working Group on Sarcopenia in Older People (EWGSOP), the International Working Group on Sarcopenia (IWGS), and the Asian Working Group on Sarcopenia (AWGS) (16–18).

Traditional methods for determining skeletal muscle mass include by anthropometry, dual-energy X-ray absorptiometry (DXA), bioresistance measurements (BIA) (19–21). In clinical practice, it is more common to use the third lumbar skeletal muscle index (L3-SMI) to determine muscle mass. The L3-SMI is calculated by obtaining a cross-section at the L3 level by computed tomography (CT) and then measuring the skeletal muscle area (SMA) by the threshold method and dividing by the square of height (m) (22). Several studies have shown that L3-SMI correlates well with patients' whole-body skeletal muscle mass, is more convenient and efficient than DXA and BIA, and has become an objective and valid evaluation index of patients' whole-body muscle mass (23, 24), which is widely used in the clinical diagnosis of sarcopenia.

### 3.2 Clinical significance of sarcopenia

In addition to other cancer-mediated consequences including anorexia, fatigue, and a decline in functional status (25), malignancy can result in a hypercatabolic state brought on by systemic inflammation and other tumor-related variables, which accelerate the progression of skeletal muscle wasting and sarcopenia (26). Therefore, cancer is usually considered the main cause of secondary sarcopenia. Depending on the tumor type, sarcopenia may be present in 20-70% of cancer patients (20) and has a significant negative impact on patient health, physical function, and quality of life (21). It has been shown that sarcopenia is highly correlated with frailty, overall survival (OS), and progression-free survival (PFS) in cancer patients (27). From a surgical oncology perspective, the presence of sarcopenia indicates that patients have limited reserves to deal with surgical stress and are more prone to complications (28, 29), longer hospital stays (30), and higher mortality (31). In other words, when sarcopenia is present in cancer patients, it often predicts a poor clinical outcome.

### 3.3 The relationship between TMT and sarcopenia

Although the cross-sectional area (CSA) of the psoas major muscle obtained by CT scan can be more accurate in assessing the muscle quality of patients, the determination of skeletal muscle quality by this muscle alone would pose certain limitations to clinical application. Recent research has demonstrated that cephalocervical skeletal muscles can also be used to detect muscle mass decrease in addition to lumbar skeletal muscles: Swartz et al. (32) demonstrated a strong correlation between the CSA of the skeletal muscles at the third cervical level (including the paravertebral and sternocleidomastoid muscles) and the CSA of the muscles at the third lumbar level. Ranganathan et al. (33) and Leitner et al. (9) further confirmed this relationship.

The above studies demonstrate that TMT can be used as an alternative assessment index for whole-body skeletal muscle mass. TMT, an imaging value that can be swiftly assessed on typical patient cranial magnetic resonance images (MRI), may therefore be a desirable approach for determining sarcopenia in neurosurgical oncology. Future clinical research would undoubtedly benefit from this, especially with patients who have brain tumors and do not frequently have abdominal CT images.

### 3.4 The role of TMT in predicting disease survival

Several research have looked into the potential connection between TMT and disease prognosis when it was discovered that it was linked to sarcopenia. Furtner et al. (11) showed that TMT is an independent predictor of survival in melanoma patients with brain metastases, and that the likelihood of patient mortality increased by 27.9% for every 1 mm reduction in TMT. Additionally, a number of other studies have demonstrated that TMT can be used as a stand-alone predictor of survival in patients with breast cancer (BC) and non-small cell lung cancer (NSCLC) with brain metastases, aiding in the clinical identification of frail patients and directing doctors in the choice of suitable therapeutic measures or in the stratification of clinical trials (10, 34, 35). Patients with primary central nervous system lymphoma (PCNSL) have a similar connection, and TMT is regarded as an independent and objective assessment measure for PCNSL patients' prognosis (36).

Thus, TMT can be used as an independent and more objective index to determine the physical status and frailty of some patients and has a predictive significance for the survival prognosis of patients.

## 4 GBM and TMT

### 4.1 Definition and characteristics of glioblastoma

Glioma makes up more than 80% of primary malignant brain tumors in adults and is the most frequent primary central nervous system tumor (37). Gliomas are categorized into grades 1 through 4 in the WHO classification of central nervous system malignancies for 2021, with grades 1 and 2 denoting low-grade gliomas and grades 3 and 4 denoting high-grade gliomas (38). Glioblastoma, commonly known as grade 4 in the WHO classification, accounts for 45.6% of primary malignant brain tumors and has an incidence rate of roughly 3.1/100,000. Despite having a low incidence rate, GBM is the most malignant and prone to relapse. With a median survival of under 2 years, the prognosis is typically poor (37). The incidence of GBM increases with age, from 0.15/100,000 in childhood to a peak of 15.03/100,000 at the age of 75-84. Its survival rate is inversely proportional to age, with a mean 5-year survival rate of <5% for GBM patients, including <2% for patients over 65 years of age (37). In addition, there is a relationship between GBM onset and gender, and studies have shown that men have a higher incidence of GBM than women (39).

There is currently little information available regarding the causes of brain tumors, and it is unclear exactly how GBM develops and spreads. Currently, the only known risk factor is exposure to high doses of ionizing radiation (40). Smoking, nutritional risk factors, and occupational risk factors have not been conclusively linked to GBM (41). Additionally, it has been proposed that allergies and infections may protect against GBM, possibly through the activation of immune surveillance pathways (40, 42, 43).

### 4.2 Clinical manifestation, treatment, and prognosis of glioblastoma

The location and size of the tumor have a significant role in determining the unusual clinical signs of GBM. Increased intracranial pressure, neurological and cognitive impairment, and seizures are typical symptoms. For example, the presence of auditory and visual disturbances indicates that the tumor is located in the temporal lobe; while tumors in the frontal region may cause personality changes in patients, thus affecting cognitive function; and large tumors with significant masses may cause gait imbalance and incontinence (44).

With the continuous development of treatment technology, the treatment of GBM has gradually become diversified. Conventional therapies include surgical resection, radiation therapy, and chemotherapy; more advanced therapies include immunotherapy, targeted therapy, viral therapy, and tumor electric field therapy. The overall prognosis of GBM is related to various factors such as the patient's age, underlying condition, tumor grade, tumor location, the extent of resection, molecular variants, treatment response, and family financial status (4). In addition, the complexity, refractoriness, and recurrence of glioma make it necessary for clinical management and multidisciplinary adjuvant treatment of patients with glioma.

### 4.3 The role of TMT in glioblastoma survival prediction

The possible usefulness of TMT as a substitute sarcopenia marker in predicting survival in GBM patients has been under increasing scrutiny in recent years. Hsieh et al. analyzed TMT measured by postoperative CT in 87 newly diagnosed GBM patients and found that TMT correlated with OS in GBM patients, with a greater median OS in those with thicker TMT than in those with thinner TMT (45). TMT was discovered by Huq et al. to be related to critical prognostic factors in GBM and to predict postoperative survival in patients with progressing GBM (46). In their analysis of 596 patients with advancing GBM using cranial MR imaging, Furtner et al. (12)found that TMT was an independent predictive factor in these patients and that those with TMT over the cutoff had improved OS and PFS; in addition, their recent study also pointed out that TMT could also be used as a reference indicator of prognosis in newly diagnosed GBM patients, and a thinning TMT suggested poor prognosis in patients (13). TMT is sensitive as an independent predictor of survival in patients with primary GBM and has the potential to predict patient survival time, according to Liu et al.'s retrospective analysis of a database of 130 primary GBM patients (47).Patients with TMT above the median group had significantly longer survival times than those with TMT below the median group. A meta-analysis conducted by Sadhwani et al. also reached the same conclusion as the former (48). A clinical study by An et al. included 177 patients with primary GBM found that the thicker group with a TMT more than the median had longer OS and PFS than the thinner group with a TMT less than the median, and the thinner group had negative associations with OS and PFS than the thicker group in matched patients. The results indicated that TMT can be a valid prognostic biomarker of clinical outcomes in patients with GBM (49). A retrospective study including 261 glioma patients conducted by Yan et al. showed that higher TMT was a protective prognostic factor for glioma patients, and patients with thicker TMT had higher OS in gliomas of different grades and IDH subtypes (50). Mi et al. presented a deep learning-based system to quantify temporalis muscle and assess its prognostic value in GBM. The results showed that temporalis muscle CSA, a non-invasive numerical prognostic biomarker, was a significant independent predictor of prognosis in patients with GBM, and patients in the above median CSA group had significantly longer OS (51). The same conclusion was reached in a study by Broen et al. The study included 328 patients with primary GBM and the final results confirmed the prognostic role of TMT as an independent survival predictor in patients with genotypic primary GBM. In addition, the study found that GBM patients at risk for sarcopenia had a significantly higher risk of early discontinuation of Stupp therapy and a significantly lower chance of receiving second-line therapy at relapse, which may be related to the fact that thinner poorer prognosis in patients with thinner TMT (52).

However, some authors have come to the opposite conclusion. In newly diagnosed GBM patients, TMT can only be used as a surrogate parameter for other epidemiological data, according to a multicenter investigation by Wende et al. (53).Their findings do not yet support the use of TMT as an independent prognostic marker.Similar conclusions were reached by Muglia et al, who found no significant correlation between TMT measured preoperatively in newly diagnosed and untreated GBM patients and patient prognosis, age at surgery, or preoperative performance status, and its validity for prognostic judgments may be limited to patients with brain metastases and recurrent or treated GBM (54). Detailed data from the references are shown in **Table 1**.

**Table 1:** Details of cited studies.

Author	Tumor(n) Primary GBM Progressive GBM	Mean TMT (mm)	Thicker group Median OS / PFS in months	HR (OS)	95%CI (OS)	*P value* (OS)
Kristin Hsieh (45)	87	−	28.30 / −	0.42	(0.22,0.82)	0.010
	−	−	− / −	−	−	−
Sakibul Huq (46)	378	9.55	13.08 / −	0.99	(0.64,1.51)	0.949
	163	9.40	8.53 / −	0.47	(0.25,0.90)	0.023
Julia Furtner (12, 13)	755	6.70	18.33 / 8.17	0.34	(0.34,0.44)	<0.010
	568	7.20	7.50 / 3.00	0.54	(0.42,0.70)	<0.010
Fang Liu (47)	130	9.31	15.60 / −	0.80	(0.69,0.92)	<0.010
	−	−	−/−	−	−	−
Geon An (49)	177	7.70	18.00/11.00	0.42	(0.30,0.58)	<0.010
	−	−	−/−	−	−	−
Ou Ying Yan (50)	261	7.50	29.00/−	0.22	(0.15,0.32)	−
	−	−	−/−	−	−	−
Ella Mi (51)	132	5.88	22.40/14.30	0.46	(0.22,0.86)	0.046
	−	−	−/−	−	−	−
Martinus Broen (52)	328	7.60	12.00/8.50	0.68	(0.50,0.92)	0.025
	−	−	−/−	−	−	−
Tim Wende (53)	335	7.00	12.00/−	1.06	(1.00,1.14)	0.070
	−	−	−/−	−	−	−
Riccardo Muglia (54)	51	8.40	20.00/−	1.34	(0.68,2.63)	0.403
	−	−	−/−	−	−	−

“-”, not mentioned.

As a result, there is still some debate about whether TMT can be used as a standalone predictor of survival in GBM patients. To further validate the utility of TMT as a prognostic reference in newly diagnosed or recurrent GBM patients, more extensive multicenter prospective studies are required in the future.

## 5 Discussion

The median patient survival for GBM, the most malignant diffuse glioma in the astrocyte spectrum, is fewer than 15 months (55). In order to provide patients with more rational individualized treatment, there is a clinical need to establish an objective and reproducible, quantifiable parameter for assessing patients’ physical status. Sarcopenia has been linked to a poor prognosis in oncology patients in previous research (56), and TMT can be utilized as a substitute marker of skeletal muscle mass to identify sarcopenia (9). The link between TMT and survival in GBM patients has come up in an increasing number of papers in recent years. Therefore, this article reviews the relevant research progress between sarcopenia, TMT, and GBM. Combined with the current state of research, there is a tendency to believe that TMT may be associated with the prognosis of GBM and can be used as a reference indicator for its survival prediction, but further validation is still needed.

### 5.1 The role of Sarcopenia as a component of frailty in predicting prognosis

While secondary sarcopenia is linked to a number of harmful events, including disease, primary sarcopenia often refers to the loss of skeletal muscle mass brought on by age. The frailty phenotype’s other elements, such as nutritional status, skeletal muscle mass, functional status, and medical comorbidities, have been demonstrated to be ultimately linked to secondary sarcopenia (57). A well-known cause of poor postoperative outcomes in surgical patients, including those with brain tumors (58), frailty is a physiological state of decreased reserve that is associated with an increased vulnerability to unfavorable clinical outcomes (59).Sarcopenia is therefore commonly used in clinics to assess patient prognosis as a part of frailty.

Sarcopenia definition and correlation with cancer prognosis is a burgeoning area for oncology study. The onset of sarcopenia is linked to a lower OS and an increased risk of patient death in both metastatic and non-metastatic breast cancer (60, 61). Additionally, studies on hepatocellular, pancreatic, ovarian, gastric, and esophageal malignancies have shown a link between sarcopenia and survival (62–66).

TMT has been shown to be correlated with aspects of frailty in recent years, including durability of grip, dietary habits, and basic functional status (8). As a result, TMT may be taken into account as part of a comprehensive preliminary fragility assessment in neurosurgical oncology for assessing patient prognosis, just as sarcopenia is used in other surgical specialties.

### 5.2 TMT’s application for the management of glioblastoma

The clinical application of TMT has obvious advantages, especially in neurosurgery. First, TMT is easy to measure; most patients with brain tumors routinely have MR images of the brain taken, and physicians can directly use MR images that are already clinically available for rapid measurement, and TMT measurement is easier to perform than the complex measurement of cross-sectional area at the L3 level. Second, TMT is not affected by muscle edema or radiation-related atrophy, for example, but only by oral disease or previous surgery (9, 12), making its clinical applicability broader. In addition, studies have shown better consistency of TMT measurements with little variability between different workers (10, 45), which is more conducive to integration into clinical routines. As mentioned previously, In order to direct the perioperative management of GBM patients and perhaps forecast patient survival cycles, the current study prefers to see TMT as a sarcopenia indicator that can be practically incorporated into the current neurosurgical workflow.

Clinicians can differentiate between patients whose physical condition and surgical tolerance are based on their preoperative TMT. Patients with a TMT above a specific threshold can undergo direct surgery, while patients with a TMT below this threshold should be recommended for a brief perioperative optimization. Although GBM surgery should not be delayed for too long, even brief perioperative optimization can compensate to some extent for the poor outcomes associated with direct surgery. Such optimization requires a multidisciplinary strategy, which includes medical co-morbidity care, physical therapy, and nutritional supplementation (67–69). Reduced TMT in patients also raises the possibility of sarcopenia. According to studies, sarcopenic GBM patients are much more likely to stop their Stupp therapy early and are significantly less likely to receive second-line therapy in the case of a relapse (52), so physicians can provide more appropriate individualized treatment plans for patients by promptly adjusting treatment measures based on changes in patient TMT. During the postoperative treatment phase, clinicians can instruct patients who need it to continue nutritional supplementation and physical therapy to maintain function through radiation and chemotherapy based on TMT. These measures may improve the quality of life and postoperative survival of patients (12, 21) and therefore warrant further research by scholars on TMT and its clinical application.

### 5.3 Potential confounders may affect the association between TMT and GBM survival

Potential confounding factors that may affect the relationship between TMT and GBM survival include the following.

The first is the baseline characteristics of the patients. Almost all current clinical retrospective studies do not include information on the ethnicity of patients, and in addition, the sex-specific thresholds for TMT used in some studies are based on standardized reference values for healthy Caucasians, thus making it impossible to rule out the possible impact of potential differences in muscle mass between races on the prognosis of TMT and GBM (52, 70). More extensive future research on patients of other races is needed to explore prognostic differences between GBM patients of different races. Patient age is also considered to be associated with GBM prognosis, but recent studies have shown no significant association between age and TMT (45, 47), breaking the previous consensus that TMT thins with age. This can be explained by the difference between biological age and actual age. Numerous studies have confirmed that biological age is thought to correlate more with death than actual age because it accurately reflects a patient’s degree of frailty, resulting in a more realistic assessment of the patient’s current physical condition (71, 72). Therefore, biological age should be more important than actual age in determining a patient’s frailty. Based on the independent prognostic effect of TMT, a patient’s biological age can be used as a stratification factor in prospective trials to assess the effectiveness of treatments targeting the patient’s degree of frailty.

The second is the patient’s response to treatment. It has been shown that patients at risk for sarcopenia (i.e., those with a TMT below the reference threshold) have a higher risk of early discontinuation of the stupp regimen and are significantly less likely to receive second-line therapy in the event of recurrent or progressive GBM, which, in turn, adversely affects the patient’s prognosis (52). In the current study, no significant correlation has been found between changes in duration and dose of corticosteroid administration and TMT values (13), but it has also been suggested that patients with recurrent GBM may experience accelerated loss of muscle mass when treated with corticosteroids (12, 73). Therefore, more detailed prospective studies are needed in the future to further investigate the interaction between TMT and steroid therapy. In addition, chemoradiotherapy itself can have an impact on TMT, with researchers noting that patients with low baseline TMT experienced TMT depletion during chemoradiotherapy and were at higher risk of death (13). Therefore, independent studies are needed to further validate the changes in TMT before and after patients receive chemoradiotherapy.

Finally, a number of comorbidities can also have an impact on the prognostic relationship between TMT and GBM. The most common is the effect of oral-related diseases. Studies have shown that patients with long-term mastication and bite force training may have thicker temporal muscles compared to the general population, while those with long-term oral diseases have thinner muscles, including the temporal muscles, due to a lack of mastication (74). Therefore, the inclusion of patients with the above conditions in studies discussing the prognostic relationship between TMT and GBM could have an impact on the results. Currently, most clinical studies do not collect a patient’s history of oral disease at the beginning, but researchers, in an effort to eliminate the interference of oral disease as much as possible, measure the patient’s bilateral TMT and take the average value for analysis. However, this effect still exists.

### 5.4 Limitations and future directions

As mentioned above, TMT is now attractive and a lot of research has been conducted. However, based on the current state of research, there are still some issues that need to be addressed. First, almost all relevant studies are currently retrospective, and most study cohorts are single-center sources with small sample sizes. Multicenter, large-scale prospective studies are needed to further validate the predictive role of TMT on prognosis. Second, standard TMT measurement methods have not been established, such as the choice of measurement equipment (CT, MRI, or ultrasound) and the optimal site for TMT measurement. Further research is needed to establish a uniform measurement method to circumvent the differences in imaging quality and reading accuracy. Third, further studies are needed to establish the TMT threshold. Currently, gender-specific TMT cutoff values have been proposed and used for grouping of study cohorts (13), which are 2.5 standard deviations lower than the standard reference population (8), consistent with the EWGSOP recommendations (75), and their external validity has been initially validated (52). It has also been suggested that separate TMT cutoffs should be used for primary and recurrent GBM, and cutoffs have been established based on the log-rank statistic of maximum selection (46). Future research may examine more complex particular to the patient TMT cutoffs that take into account comorbidities, gender, race, and other variables. Fourth, TMT may have a strong relationship with young age, which is also connected to a better prognosis, IDH mutations, and high levels of MGMT promoter methylation (48). Therefore, future attempts at multivariate analysis using other pertinent clinical and molecular prognostic markers are suggested (for example, age, preoperative performance status, IDH mutation status, and MGMT promoter methylation status).

In the future, TMT measurement may be combined with deep learning models, artificial intelligence, etc. to automate TMT measurement from MR images and integrate it into clinical workflows. This will simplify clinical operations while improving measurement accuracy and efficiency and reducing measurement heterogeneity and will allow physicians to dynamically monitor TMT changes during patient follow-up to determine patient prognosis.

## 6 Conclusion

A potential prognostic factor, TMT appears to be a good substitute marker of skeletal muscle volume and function. The amount of research on the connection between GBM and TMT is expanding. The link between TMT, sarcopenia, and physical state in GBM patients has to be investigated further.

## Author contributions

Study concept and design: XL and JT; Visualization: PY and JT; Writing the manuscript: JT, ZD, PY; Investigation: all authors. All authors contributed to the article and approved the submitted version.
