# Supplementary material for: Advances in the relationship between temporal muscle thickness and prognosis of patients with glioblastoma: a narrative review
Source: Front Oncol. 2023 Sep 13;13:1251662. doi: 10.3389/fonc.2023.1251662 (PMC10525700; doi:10.3389/fonc.2023.1251662)
Supplement: Supplementary file 1 [file Table_1.docx]

| Database: PubMed | |
| --- | --- |
| #1 | “GBM”[All fields] |
| #2 | “Glioblastoma”[Mesh] |
| #3 | “Glioma*”[Mesh] |
| #4 | “glioblastoma *”[All fields] |
| #5 | “glioblastoma multiforme”[All Fields] |
| #6 | #1 OR #2 OR #3 OR #4 OR #5 |
| #7 | “sarcopenia” [Mesh] |
| #8 | exp sarcopenia/ |
| #9 | sarcopeni*[Title/Abstract] |
| #10 | ((muscle[tiab] OR muscular[tiab]) AND (atroph*[tiab] OR wasting*[tiab] OR weak*[tiab] OR loss*[tiab] OR depletion*[tiab])) |
| #11 | temporal muscle [Title/Abstract] |
| #12 | #7 OR #8 OR #9 OR #10 OR #11 |
| #13 | #6 AND #12 |
| Database: Embase | |
| 1 | 'exp sarcopenia' OR (exp AND ('sarcopenia'/exp OR sarcopenia)) |
| 2 | sarcopeni*.ti,ab. |
| 3 | ((muscle or muscular) adj2 (atroph* or wasting* or weak* or loss* or depletion*)).ti,ab. |
| 4 | exp temporal muscle/ |
| 5 | 1 OR 2 OR 3 OR 4 |
| 6 | glioblastoma.mp. or exp glioblastoma/ |
| 7 | gbm.mp. |
| 8 | (high grade adj2 glioma).mp. |
| 9 | hgg.mp. |
| 10 | (grade IV adj2 glioma).mp. |
| 11 | (grade IV adj2 astrocytoma).mp. |
| 12 | 6or 7 or 8 or 9 or 10 or 11 |
| 13 | 5 and 12 |
| Database: Medline | |
| #1 | exp Glioblastoma/ or glioblastoma*.mp. |
| #2 | gbm.mp. |
| #3 | high grade glioma.mp. |
| #4 | hgg.mp. |
| #5 | (glioma adj2 grade IV).mp. |
| #6 | (astrocytoma adj grade IV).mp. |
| #7 | 1 or 2 or 3 or 4 or 5 or 6 |
| #8 | exp sarcopenia/ |
| #9 | sarcopeni*.ti,ab. |
| #10 | ((muscle or muscular) adj2 (atroph* or wasting* or weak* or loss* or depletion*)).ti,ab. |
| #11 | exp temporal muscle/ |
| #12 | 8 OR 9 OR 10 OR 11 |
| #13 | 7 and 12 |
